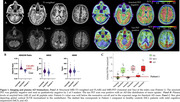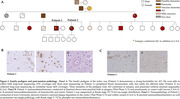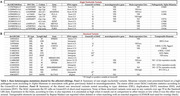# Genetic Analysis of an Amyloid PET‐Negative Autopsy‐Confirmed Alzheimer's Pedigree

**DOI:** 10.1002/alz.093931

**Published:** 2025-01-09

**Authors:** Lia Talozzi, Andrés Peña‐Tauber, Tanner D. Jensen, Edward N. Wilson, Christina B. Young, Ilaria Stewart, Inma Cobos, Kristen Vallejo, Marco M Hefti, Yann Le Guen, Kennedy Williams, Hillary Vossler, John Gorzynski, Katrin I. Andreasson, Elizabeth Mormino, Euan Ashley, Michael D Greicius

**Affiliations:** ^1^ Stanford University, School of Medicine, Stanford, CA USA; ^2^ Stanford University School of Medicine, Stanford, CA USA; ^3^ Stanford University, Stanford, CA USA; ^4^ University of Iowa, Iowa City, IA USA

## Abstract

**Background:**

Alzheimer's disease (AD) is the most common form of dementia. Neuropathologically, AD stands out as a mixed proteinopathy. Beta‐amyloid and tau biomarkers can now add in‐vivo support to the AD diagnosis. Rarely, a patient with AD confirmed at autopsy may have a negative amyloid PET. Here we describe a pedigree in whom the index case was amyloid PET‐negative. We performed genetic sequencing of two affected siblings to identify a set of candidate single nucleotide variants (SNVs) and structural variants (SVs) associated with disease and rare in healthy older controls from several large genetic databases.

**Method:**

We performed long‐read sequencing (LRS) to comprehensively evaluate both SNVs and SVs. The index case, an APOE3/E4 male, developed memory trouble at 68 that progressed to probable AD at 72. Notably (Figure 1) his amyloid‐PET scan, which was confirmed to be of high technical quality, was negative. His tau PET scan was positive and his plasma Abeta42/40 was in the expected range for Stanford AD patients. His family history is extensive and suggestive of an autosomal dominant pattern of late‐onset AD (Figure 2). The patient's sister was diagnosed with AD at 62. We filtered their shared heterozygous SNVs keeping those with a minor allele frequency < 0.01 in gnomAD and that were not present in any ADSP healthy controls (HC) over 70y.o. (N=19771;62.1%females;81±6.4y.o). Shared SVs were kept as candidates if they were not seen in any Stanford LRS HC (N=95;59%females;72±1.5y.o.)

**Result:**

Neuropathological analysis revealed that both siblings met the A3B3C3 criteria for AD and had cerebral amyloid angiopathy. Among rare mutations on genes expressed in the brain (Table 1), a novel missense in ADNP (Activity‐Dependent‐Neuroprotector‐Homeobox), and a 267bp deletion on TET1 (Tet‐Methylcytosine‐Dioxygenase) were identified. The 3426bp insertion on ATP8A2 (ATPase‐Phospholipid‐Transporting‐8A2) was the longest, rare insertion found. Additionally, a large duplication (∼20Kbp) and inversion (∼160Kbp) were observed.

**Conclusion:**

This study highlights an unusual AD pedigree and emphasizes the potential utility of LRS in identifying causal SNVs and SVs. In future work we plan to perform additional amyloid stains to understand why the index case PET was amyloid‐negative. We are also assessing additional family members to improve our ability to identify a causal mutation.